# Influence of Biological Maturation on the Career Trajectory of Football Players: Does It Predict Elite Success?

**DOI:** 10.3390/jfmk10020153

**Published:** 2025-04-30

**Authors:** Saül Aixa-Requena, Albert Gil-Galve, Alejandro Legaz-Arrese, Vicenç Hernández-González, Joaquín Reverter-Masia

**Affiliations:** 1 Human Movement Research Group (RGHM), University of Lleida, Plaça de Víctor Siurana, 25003 Lleida, Spain; saul.aixa@udl.cat (S.A.-R.); joaquim.reverter@udl.cat (J.R.-M.); 2 Physical Education and Sport Section, University of Lleida, Av. De l’Estudi General, 25001 Lleida, Spain; 3 Director of Youth Football at Real Madrid, Deporivo La Coruña, 15011 La Coruña, Spain; albertogilgalve@gmail.com; 4 Faculty of Health and Sport Sciences, University of Zaragoza, 22002 Huesca, Spain; alegaz@unizar.es

**Keywords:** biological maturation, talent selection, youth football, sports development, elite athletes

## Abstract

**Background:** Early-maturing players tend to have physical advantages during formative stages, but it remains unclear whether these advantages translate into long-term professional success. This study examines how biological maturation influences participation and career trajectories in youth football. **Methods:** Anthropometric and competitive data were collected from 47 players (13.53 ± 1.08 years) in a top-tier academy during the 2010–2011 season. The maturation status was assessed using the Tanner–Whitehouse II RUS method, and the career outcomes were tracked in 2024–2025. **Results:** Early-maturing players showed higher anthropometric values and greater participation. However, late maturers were more likely to reach professional football (*p* = 0.003), with all players competing in the top five European leagues belonging to the late-maturing group. **Conclusions:** Early maturation does not guarantee professional success. Strategies such as bio-banding and personalized training can reduce biases and support talent development, highlighting the need for a more holistic approach to player evaluation.

## 1. Introduction

Talent development in youth football is a complex process influenced by physical, technical, tactical, and psychological factors [1]. Among these, biological maturation has been identified as a key element in the identification and development of young football players, particularly in elite settings [2].

Early-maturing players tend to present physical advantages during formative stages, which facilitates their selection into competitive teams [3,4]. These advantages are reflected in better performance in speed, strength, and aerobic endurance [5], increasing their chances of participating and remaining in professional academies, although this can generate biases in the selection process [6]. However, these advantages do not always translate into long-term success, as other compensatory skills become more relevant over time [7].

Maturation also influences the ability to adapt to training loads and the risk of injury. While early maturers can tolerate higher loads at younger ages, they also face a greater risk of overload [8]. In contrast, late maturers tend to develop more gradually, which may enhance their athletic longevity [9].

To address these biases, strategies such as bio-banding have been proposed, which group players according to their biological maturation rather than chronological age, allowing for a fairer assessment of talent [10,11].

Although the influence of maturation on youth performance has been widely studied, there is limited longitudinal research examining its impact on professional success. Some studies suggest that the advantages associated with early maturation tend to diminish over time [12,13], while others highlight the relevance of biological age in performance progression [14].

In this context, the present study aims to analyze whether biological maturation during adolescence predicts adult performance in players trained in an elite academy. Through the longitudinal monitoring of a cohort of youth and U16 football players, the objective is to determine whether early maturation represents a sustained advantage or if its influence is surpassed by other factors throughout athletic development.

## 2. Materials and Methods

### 2.1. Participants

This study included 47 male players (13.53 ± 1.08 years) (24 in the U16 category and 23 in the U14 category) involved during the 2010–2011 season in a top-tier football academy belonging to a professional Spanish football club at the international level. All participants had been registered with and trained exclusively in this academy since pre-pubertal age. The sample was divided according to the competition category: 24 players in the U16 category and 23 players in the U14 category. Informed consent was obtained from all the subjects involved in the study.

### 2.2. Procedures

Anthropometric data were collected, including height, weight, and body mass index (BMI), with the aim of analyzing their relationship with biological maturation. Height was measured in a bipedal position using a precision stadiometer in millimeters, while weight was determined using a calibrated digital scale. BMI was calculated using the following formula: weight (kg)/height^2^ (m^2^).

Chronological age was subsequently assessed based on the birth date recorded in the players’ official documents, and biological age was evaluated at the “Clinica Cemtro” (Madrid, Spain) in February 2011 using the Tanner–Whitehouse II (TW2-RUS) method, which estimates bone maturation based on the development of 13 bones in the dominant hand and wrist [15].

The Tanner–Whitehouse II (TW2-RUS) method is one of the most widely used tools in the sports field to assess bone maturation and determine the biological age of young athletes. This method is based on the analysis of 13 bones in the hand and wrist, assigning specific scores to each one according to its degree of development, which allows for a precise estimation of maturational status [16,17].

Participants were classified into three biological maturation categories based on the difference between their bone age and chronological age. Early maturers were those whose biological age exceeded their chronological age by more than 0.51 years, on-time maturers were those whose biological age was within a ±0.50-year range of their chronological age, and late maturers were those whose biological age was more than 0.51 years below their chronological age. This margin of ±0.51 years accounts for the error associated with the estimation of bone age using the TW2-RUS method [12].

The competitive participation variables were recorded, including the minutes available and the minutes played by each footballer during the 2010–2011 season, allowing the impact of maturation on competitive participation to be evaluated. The available minutes were defined as the total minutes in which the player was eligible for selection by the coaching staff, excluding matches missed due to injury, suspension, or tactical decisions. The minutes played corresponded to the total minutes each player competed in official matches. Based on these data, the percentage of playing time was calculated, defined as the proportion of minutes played relative to the available minutes, thus estimating the coach’s confidence in the player’s selection.

To analyze the players’ career trajectories in adulthood, information was collected in 2024 on the category in which they were competing during the 2024–2025 season. Data were obtained from publicly available databases commonly used by the club for player monitoring.

A player was considered to have reached professional football if, during the 2024–2025 season, they were actively competing in one of the five major European leagues (Spanish LaLiga, English Premier League, German Bundesliga, Italian Serie A, and French Ligue 1) or in the Spanish Segunda División. Previous playing history was not taken into account; only the category in which they were competing at the time of data collection was considered, ensuring that they had not only reached these levels but had also remained in them.

### 2.3. Statistical Analysis

All analyses were conducted using IBM SPSS Statistics v.26 software (IBM Corp., Armonk, NY, USA). The descriptive statistics for the sample included means and standard deviations for the continuous variables and percentages for the categorical variables. The normality of the variables was tested using the Shapiro–Wilk test, and the homogeneity of variances was tested using Levene’s test.

A descriptive analysis was performed to assess the distribution of players according to their biological maturation overall, by category, and among those who achieved success in their professional careers.

To evaluate differences between biological maturation groups (early, on-time, and late maturers) in the anthropometric and competitive participation variables, a one-way analysis of variance (ANOVA) was conducted. Effect size was calculated using eta squared (η^2^) to determine the magnitude of the observed differences. According to the established criteria [18], an η^2^ value less than 0.1 was considered a trivial effect, between 0.1 and 0.29 a small effect, between 0.30 and 0.49 a medium/moderate effect, and greater than 0.50 a large effect. In cases of significant differences (*p* < 0.05), post hoc comparisons were performed using the Tukey HSD test to identify the specific groups with significant differences.

Additionally, the relationship between biological maturation and the competitive level reached in the 2024–2025 season was analyzed using a chi-square test with continuity correction (χ^2^cc) to assess the association between these two variables, with the effect size measured using Cramer’s V. Following Cohen’s guidelines for χ^2^ tests [19] and adjusting the benchmarks to two degrees of freedom (df = 2), an effect size was considered small if V ≥ 0.07, moderate if V ≥ 0.21, and large if V ≥ 0.35.

Using G*Power 3.1 (α = 0.05, power = 0.80), we found that a one-way ANOVA with three maturation groups would require 26 players to detect a moderate effect (η^2^ ≈ 0.30) and 14 players for a large effect (η^2^ ≈ 0.50), while a 3 × 2 χ^2^ test would need 108 players for a moderate association (w = 0.30, V ≈ 0.21) and 39 players for a large one (w = 0.50, V ≈ 0.35). Our current sample (*N* = 47), therefore, satisfies the minimum for moderate-to-large effects in the ANOVA and for large effects in the χ^2^ analysis but remains underpowered for detecting moderate χ^2^ effects.

This study was approved by the Clinical Research Ethics Committee of the Catalan Sports Council (30/CEICGC/2020).

## 3. Results

### 3.1. Differences in Anthropometric Characteristics

Early-maturing players showed higher chronological age, bone age, height, weight, and BMI compared to those with normal and late maturation (Table 1). Progressively, players with normal maturation also exhibited higher values than late-maturing players for these variables.

The analysis of variance (ANOVA) (Table 2) revealed significant differences between maturation groups in age, height, weight, and BMI (*p* < 0.05). Post hoc comparisons indicated that early maturers had significantly higher values than late maturers in all of these measures, whereas differences between on-time maturers and late maturers were less consistent.

### 3.2. Competitive Participation

In the overall sample, the percentage of minutes played was higher among early-maturing players, followed by on-time maturers, with the lowest percentage among late maturers (Table 1), although the differences were not statistically significant (*p* = 0.078) (Table 2). A similar pattern was observed in both the U16 and U14 categories, with late maturers accumulating fewer minutes played, but without significant differences (*p* = 0.056; *p* = 0.101).

### 3.3. Career Trajectory and Access to Elite Football

In the 2024–2025 season, seven players reached and remained in professional football within the five major European leagues and the Spanish Segunda División, four of whom played in the top-tier European leagues (Spanish La Liga, English Premier League, German Bundesliga, Italian Serie A, and French Ligue 1).

A total of 14.9% of the sample reached professional football. By maturation status, 5.6% of early maturers, 12.5% of on-time maturers, and 30.8% of late maturers reached this level (*p* = 0.003, Table 2).

All of the players who competed in the five major European leagues belonged to the late-maturing group. No early or on-time maturers reached this competitive level (Table 3).

## 4. Discussion

The results of this study challenge the widely accepted notion that early maturation provides a decisive advantage in accessing professional football. Although early-maturing players are often favored during formative stages due to their physical superiority, our findings show that the players who reached the major European leagues in the 2024–2025 season predominantly belonged to the late-maturing group.

While late maturers recorded a lower percentage of minutes played, these differences were not statistically significant, suggesting that their reduced participation was not a limiting factor for their development. In fact, some of these players had high competitive participation, possibly due to other technical, tactical, or psychological qualities.

These findings are consistent with previous research indicating that the physical advantages of early maturers tend to diminish over time [7]. In contrast, late maturers may develop compensatory skills (such as greater technical ability, cognitive capacity, or psychological resilience) that enhance their performance at the highest level [11,20]. This reinforces the importance of prioritizing technical and tactical indicators over morphological characteristics in talent identification [21].

Moreover, late-maturing players may develop greater psychological resilience and better adaptability to adverse situations, which could explain their success in transitioning to professional football [9]. Competing against physically more developed opponents from an early age may enhance key skills such as decision-making under pressure and creativity in gameplay, both critical factors in high-performance football.

From a psychological perspective, late-maturing players may develop enhanced emotional regulation and mental toughness due to the early challenges they face in competing against more physically developed peers [22]. This increased exposure to adversity may foster improved stress management and problem-solving skills, which become highly valuable in professional environments where athletes face high-pressure situations and complex tactical demands. Furthermore, late maturers may also develop stronger leadership skills and enhanced communication on the field, as they are forced to rely more on strategy and teamwork rather than physical dominance during their formative years [23]. The ability to remain composed and adaptable under pressure has been linked to improved performance consistency at elite levels, suggesting that psychological resilience may serve as a key factor in long-term athletic success [24].

From a developmental perspective, ensuring an equitable distribution of playing time is essential to support the growth of all player profiles [25]. In this study, institutional measures, such as guaranteeing a minimum number of minutes per player, were key to providing late-maturing footballers with competitive opportunities. Notably, the only four players from this cohort currently competing in the five major European leagues belonged to the late-maturing group, highlighting the positive impact of these developmental policies. This finding aligns with the proposal that early-maturing players may be exposed to higher training volumes and intensities from a young age [26], increasing their risk of injury [27,28] and potentially affecting their long-term development [8]. In contrast, late-maturing players may benefit from a more gradual developmental trajectory, allowing them to reach peak performance at a later stage in their careers.

This pattern reflects the broader findings in athlete development research, which suggest that late-maturing athletes may experience longer career longevity due to reduced physical stress and lower rates of overuse injuries during adolescence [29]. Studies have shown that early-maturing athletes are often subjected to higher training loads and competitive demands, which can lead to burnout and chronic injuries later in their careers [30]. In contrast, late-maturing athletes, who develop more gradually, may avoid these pitfalls and sustain peak performance for a longer period. This aligns with evidence from other sports, such as basketball and swimming, where late physical maturation has been linked to improved career longevity and greater consistency in high-performance settings [31]. Adopting training models that accommodate individual maturation status could, therefore, enhance long-term athletic success and reduce the risk of premature career termination [2,32,33].

Another important factor is the influence of competitive level on player progression. The increased physical demands and tactical complexity at higher levels may favor players with enhanced technical and cognitive skills rather than purely physical attributes. Late-maturing players who have adapted to competing against more physically developed peers may, therefore, have a strategic/psychologic advantage in high-performance environments [34]. This suggests that talent identification programs should integrate not only biological maturation but also technical and tactical development when evaluating long-term potential.

Regarding talent identification processes, biological maturation has been shown to carry more weight than relative age in selection within academies [35,36]. This underscores the need to adopt strategies that minimize maturation biases. Among these strategies, bio-banding has proven effective by grouping players according to their biological maturation rather than chronological age, allowing for a fairer assessment of potential talent [11]. Complementing this approach with technical, tactical, and psychological evaluations could optimize the identification and development of talent in the long term [37].

### Limitations and Future Directions

This study is limited to a single academy, which restricts the generalizability of the findings beyond elite-level environments. The small number of professional players makes it difficult to draw firm conclusions, and technical, psychological, or contextual factors were not considered. Additionally, the estimation of biological maturation may have a margin of error.

Access to suitable data imposed further constraints on the study. Our cohort of 47 athletes comprises every player enrolled in the high-performance program of a club that was—and still is—one of the premier football organizations in Spain during the 2010–2011 season. Because comparable longitudinal bone-age records from other elite academies were unavailable, the sample could not be enlarged. In addition, biological maturation was captured with only a single TW2-RUS scan at baseline, which prevents us from modeling individual tempo-by-timing interactions or the psychological adjustments that accompany pubertal change factors [38], which could influence sport dropout or other related outcomes. No follow-up assessments were conducted to document adult height or body mass, so we cannot determine whether pubertal timing produced lasting differences in stature or BMI. Nevertheless, longitudinal work shows that late-maturing boys usually reach adult sizes similar to their earlier-maturing peers [2,13]. Future studies should employ serial bone-age assessments or peak-height-velocity modeling to capture individual maturation tempos and their potential psychological and performance consequences.

Although all but one player were of Spanish–European ancestry, we collected no genomic or epigenetic information. This is a relevant shortcoming because ethnic background can influence the timing and tempo of biological maturation [39]. For example, a recent systematic review found African youths to display bone-age advances of roughly four to six months relative to European standards [40]. Without such data, we cannot disentangle whether the developmental patterns we observed are specific to this population or reflect broader genetic influences. Future studies should, therefore, incorporate ancestry markers or polygenic maturity scores to verify whether the links between maturation status and career outcomes are consistent across genetic backgrounds.

Despite the modest sample size, the large effect sizes observed—together with the exceptional competitive level of both the players and their training environment—suggest that the findings remain informative and worthy of consideration. Future studies should include larger and more diverse samples, consider additional variables related to talent development, and analyze the impact of strategies such as bio-banding in different competitive contexts.

Future research should explore the long-term career trajectories of both early and late-maturing players to better understand the factors influencing professional success and career longevity. Tracking the performance of late-maturing players over time could clarify whether their compensatory skills—such as technical ability and psychological resilience—become decisive at the highest levels of competition.

Further studies should evaluate how training loads and competitive intensity should be adjusted according to maturation status to enhance long-term performance and reduce injury risk. Comparing the career outcomes of players involved in bio-banding programs versus those in traditional age-based systems could also provide valuable insights into the effectiveness of maturation-based selection strategies.

Additionally, future research should adopt a more holistic approach to talent identification and development, integrating physical, technical, tactical, and psychological evaluations to create more effective pathways for player development.

## 5. Conclusions

Biological maturation is not a decisive predictor of success in professional football. Although early-maturing players have initial physical advantages, late-maturing players may compensate with other types of skills. Reducing selection biases through strategies such as bio-banding and adjusting training loads based on individual maturation is essential. Long-term development should take precedence over immediate performance.

## Figures and Tables

**Table 1:** Anthropometric and competitive characteristics of the participants.

Group	Maturation	*n*	Chronological Age (Years)	Bone Age (Years)	Height (cm)	Weight (kg)	BMI (kg/m^2^)	Available Time (min)	Playing Time (min)	Time Played (%)
Global	Early	18	14.2 ± 0.9 ^c^	15.8 ± 1.5 ^c^	167 ± 8 ^c^	58.1 ± 7.8 ^c^	20.7 ± 1.2 ^c^	2040 ± 320	1051 ± 289	53 ± 10
	On-Time	16	13.4 ± 1.1	13.3 ± 1.1	159 ± 9	47.3 ± 10.5	18.4 ± 2.0	1980 ± 310	1109 ± 298	58 ± 17
	Late	13	12.9 ± 0.9 ^b^	11.8 ± 1.1 ^ab^	149 ± 7 ^ab^	40.1 ± 7.1 ^b^	17.8 ± 1.8 ^b^	1800 ± 290	790 ± 328 ^ab^	45 ± 19
	Total	47	13.5 ± 1.1	14.0 ± 2.1	159 ± 11	49.5 ± 11.3	19.1 ± 2.1	1940 ± 315	996 ± 325	52 ± 16
U16	Early	14	14.6 ± 0.3	16.5 ± 0.8 ^c^	170 ± 6	61.1 ± 5.6	21.1 ± 1.0	2085 ± 309	1136 ± 264	55 ± 10
	On-Time	7	14.5 ± 0.3	14.5 ± 0.3	167 ± 6	56.2 ± 9.9	19.9 ± 2.1	2262 ± 164	1127 ± 282	50 ± 11
	Late	3	14.3 ± 0.4	13.2 ± 0.3 ^ab^	155 ± 4 ^ab^	47.1 ± 2.8 ^b^	19.7 ± 0.6	2080 ± 366	817 ± 164	39 ± 2
	Total	24	14.5 ± 0.3	15.5 ± 1.4	167 ± 8	57.9 ± 8.1	20.6 ± 1.5	2136 ± 282	1093 ± 272	51 ± 11
U14	Early	9	12.6 ± 0.2	13.4 ± 0.3 ^c^	157 ± 6	47.7 ± 4.6	19.3 ± 0.5	1627 ± 119	754 ± 138	46 ± 7
	On-Time	4	12.5 ± 0.3	12.4 ± 0.4	152 ± 3	40.3 ± 3.1	17.3 ± 1.0	1671 ± 137	1092 ± 329	65 ± 18
	Late	10	12.5 ± 0.3	11.1 ± 0.6 ^ab^	148 ± 7 ^b^	38.0 ± 6.7 ^b^	17.2 ± 1.6 ^b^	1687 ± 166	781 ± 370	46 ± 22
	Total	23	12.5 ± 0.3	12.2 ± 1.0	151 ± 7	40.6 ± 6.1	17.6 ± 1.4	1670 ± 143	889 ± 349	53 ± 20

Note. Values are presented as mean ± SD. Superscripts denote pairwise differences (Tukey post hoc, *p* < 0.05): ^a^ Late vs. On-time; ^b^ Late vs. Early; ^c^ Early vs. On-time.

**Table 2:** Inferential statistical results according to biological maturation.

Variable		Global			U16			U14	
	F	*p*	η^2^	F	*p*	η^2^	F	*p*	η^2^
Chronological Age (years)	7.37	0.002 **^,bc^	0.251	1.88	0.178	0.152	0.58	0.573	0.054
Bone Age (years)	34.98	<0.001 ***^,abc^	0.630	47.99	<0.001 ***^,abc^	0.820	29.42	<0.001 ***^,abc^	0.776
Height (cm)	16.73	<0.001 ***^,abc^	0.432	6.93	0.005 **^,ab^	0.398	3.83	0.039 *^,b^	0.277
Weight (kg)	17.03	<0.001 ***^,bc^	0.436	5.23	0.014 *^,b^	0.332	5.02	0.017 *^,b^	0.334
BMI (kg/m^2^)	13.74	<0.001 ***^,bc^	0.385	2.70	0.091	0.204	4.06	0.033 *^,b^	0.289
Available Time (min)	2.71	0.078	0.112	3.32	0.056	0.240	2.59	0.101	0.214
Playing Time (min)	4.33	0.019 *^,ab^	0.168	1.91	0.173	0.154	2.40	0.118	0.202
Time Played (%)	2.71	0.078	0.112	3.32	0.056	0.240	2.59	0.101	0.214
Players who reached elite level	11.44	0.003 **	0.493	15.27	<0.001 ***	0.798	2.85	0.241	0.352

Note. A one-way ANOVA was used to assess the association between maturation status (early, on-time, and late) and the analyzed variables, with effect size measured using η^2^. For the “Players who reached elite level”, a chi-square test with continuity correction (χ^2^cc) was performed, with effect size measured using Cramer’s V. * *p* < 0.05; ** *p* < 0.01; *** *p* < 0.001. Superscripts denote pairwise differences (Tukey post hoc, *p* < 0.05): ^a^ Late vs. On-time; ^b^ Late vs. Early; ^c^ Early vs. On-time.

**Table 3:** Anthropometric and competitive characteristics of professional players in 2024–2025.

Player	Maturation	Group	League	Chronological Age (Years)	Bone Age (Years)	Height (cm)	Weight (kg)	BMI (kg/m^2^)	Available Time (min)	Playing Time (min)	Time Played (%)
A	Late	U14	Big Five	12.1	11.5	147	35.0	16.3	1680	1398	83
B	Late	U14	Big Five	12.4	11.5	145	38.8	18.5	1680	1450	86
C	Late	U16	Big Five	13.9	13.0	152	46.0	19.9	1680	630	37
D	Late	U16	Big Five	14.3	13.0	154	45.1	19.0	2400	996	41
E	On-Time	U14	Spanish 2nd	12.3	12.5	155	44.1	18.3	1750	1196	68
F	On-Time	U16	Spanish 2nd	14.8	15.0	165	52.9	19.4	2400	1479	61
G	Early	U14	Spanish 2nd	12.5	13.5	164	51.4	19.2	1750	875	50

Note. The “Big Five” refers to the five major European leagues: Spanish LaLiga, English Premier League, German Bundesliga, Italian Serie A, and French Ligue 1. Spanish 2nd refers to “Segunda divisón española”.

## Data Availability

Data are available on request from the authors.
